# Prevalence and Implications of Occult Hepatitis B Virus Infection Among Blood Donors in Saudi Arabia: A Systematic Review and Meta-Analysis

**DOI:** 10.3390/diagnostics15233004

**Published:** 2025-11-26

**Authors:** Wajnat A. Tounsi

**Affiliations:** Department of Medical Laboratory Sciences, Faculty of Applied Medical Sciences, King Abdulaziz University, Jeddah 21589, Saudi Arabia; wtounsi@kau.edu.sa; Tel.: +966-546-336-494

**Keywords:** occult HBV infection, transfusion-transmitted infections, blood donor screening, hemovigilance, blood safety

## Abstract

**Background**: Hepatitis B virus (HBV) remains a transfusion-transmissible infection of global concern. While mandatory screening for hepatitis B surface antigen (HBsAg) has reduced overt infections, occult hepatitis B infection (OBI) poses ongoing risk. This meta-analysis aimed to estimate the pooled prevalence of anti-HBc, HBsAg, and OBI in Saudi blood donors, as well as to assess regional variations and temporal trends. **Methods**: A systematic meta-analysis was conducted using nine studies published between 2013 and 2024, encompassing a total of 87,820 blood donors. Prevalence was pooled using random-effects models with logit transformation and Hartung–Knapp adjustment. Heterogeneity was quantified with I^2^ and τ^2^. Subgroup analyses examined geographic regions; meta-regression assessed publication year. Publication bias was evaluated with Egger’s regression; sensitivity analyses tested robustness. **Results**: The pooled prevalence of anti-HBc was 5% (95% CI: 3–7%), HBsAg was 0.46% (95% CI: 0.31–0.69%), and OBI was 0.12% (95% CI: 0.03–0.39%). High heterogeneity was observed (I^2^ = 90.6–98.9%). Subgroup analyses revealed regional disparities, and meta-regression showed no significant temporal trends. **Conclusions**: The findings suggest that past HBV exposure remains relatively common among Saudi blood donors, while current active infection and OBI are infrequent. These results support continued enhanced screening, including anti-HBc and nucleic acid testing (NAT), to ensure transfusion safety. The lack of a national hemovigilance system that monitors transfusion-transmitted infections among recipients represents a critical gap in Saudi Arabia’s transfusion safety framework. Addressing this gap is essential to fully assess residual risks, evaluate the real-world effectiveness of screening policies, and align with global best practices in blood safety.

## 1. Introduction

Blood transfusion is a critical life-saving intervention, yet it presents a risk for transmission of transfusion-transmissible infections (TTIs), including HBV. Hepatitis B virus (HBV) remains a major global public health concern, with an estimated 257–296 million people living with chronic infection and around 820,000 annual deaths [1,2].

Occult hepatitis B infection (OBI) is defined as detectable HBV DNA in the absence of HBsAg. OBI remains a clinically silent yet significant threat to the safety of blood transfusion services worldwide, which can lead to reactivation in immunosuppressed patients, progression to chronic liver disease, and even hepatocellular carcinoma [3,4,5,6]. The mechanisms underlying OBI often involve suppressed HBV replication due to host immune control rather than viral mutations alone; while molecular and immunological factors, including HBV covalently closed circular DNA (cccDNA) persistence and integration into host genomes, contribute to the OBI phenotype [7,8]. Studies show that OBI infectivity is shaped by both donor and recipient immunity, in which higher donor HBV DNA and low/absent donor anti-HBs increase the risk of infection, while recipient anti-HBs (±anti-HBc) is protective. OBI units containing anti-HBs are generally far less infectious, although low anti-HBs may be overcome by higher viral loads [9].

OBI prevalence varies globally and is notably higher in anti-HBc-positive individuals and in settings where nucleic acid testing (NAT) is not routinely implemented. This virological profile is particularly concerning in blood donation contexts, where OBI carriers may contribute to viral transmission. A recent study estimated the global OBI prevalence among HBsAg-negative blood donors at approximately 0.2%, rising to 6.2% among those positive for anti-HBc [10]. Geographic variability reflects differences in socioeconomic conditions, screening protocols, and underlying viral genetics. In Saudi Arabia, HBV is overwhelmingly genotype D (~97\%), predominantly D1, mirroring patterns across the Middle East. This genotype context should be considered when examining OBI transfusion-risk data from non-D-dominant settings [11].

International data show that combining anti-HBc screening with individual donation NAT reduces transfusion-transmitted HBV infection. In Japan, analysis of about 41 million donations reported an incidence of ~0.19 per million after the implantation of NAT, with OBI-related HBV transmission effectively eliminated after tightening the anti-HBc threshold [12]. At the same time, recent works have highlighted that confirming NAT-positive/HBsAg-negative donations often requires enhanced algorithms (repeated discriminatory NAT and serial NAT) because many true OBI cases show intermittent, very low-level viremia [13,14]. Also, seronegative OBI is invisible to serology because viremia is low/intermittent; therefore, NAT is relied on for detection [15]. In response, the Ministry of Health in Saudi Arabia made NAT screening for HBV, HCV, and HIV compulsory for all blood donors nationwide by the end of 2008 [16]. Since then, all blood banks and hospital transfusion services have been required to perform NAT on every blood donation, alongside standard serological tests such as HBsAg, anti-HBc, anti-HCV, and HIV antibodies.

In Saudi Arabia, several regional studies have highlighted the relevance of OBI. Reported anti-HBc positivity rates range from 6% in Makkah [17] to over 6% in Qassim and Al-Majma’ah [18,19]. In Riyadh, HBV DNA was detected in 8.6% of anti-HBc positive, HBsAg-negative donors [20], while Aseer reported 1.09% NAT positivity in similar cases [21]. A larger cohort in the same region revealed a 0.057% OBI prevalence [22]. In Jazan, NAT positivity reached 0.4%, with a 7.3% anti-HBc rate [23]. Al-Baha and Aseer reported HBV DNA detection rates of 0.4% and 0.3%, respectively [24,25].

Despite advancements in donor screening, a comprehensive understanding of transfusion safety remains incomplete without monitoring post-transfusion outcomes. A critical limitation in the current Saudi transfusion system is the absence of a national hemovigilance framework focused on recipients [26]. Existing surveillance mechanisms emphasize donor safety but do not track TTIs, including OBI, among recipients. This gap limits efforts to assess the real-world impact of screening interventions and to identify ongoing transmission risks. Establishing a recipient-centered hemovigilance system is essential to closing this loop and aligning with WHO-recommended best practices [27].

To date, no systematic review has quantified the national burden of OBI among blood donors in Saudi Arabia. This study primarily aims to estimate the pooled prevalence of OBI, and assess the prevalence of related HBV markers (anti-HBc and HBsAg). The findings provide critical insight into residual transfusion risks, informing national transfusion safety practices and supporting the development of recipient-focused hemovigilance systems.

## 2. Materials and Methods

### 2.1. Protocol and Registration

This systematic review and meta-analysis aimed to estimate the pooled prevalence of HBV seromarkers, including anti-HBc, HBsAg, and OBI, among blood donors in Saudi Arabia. The study protocol was registered in a publicly accessible registry [CRD420251060519] and conducted in accordance with the Preferred Reporting Items for Systematic Reviews and Meta-Analyses (PRISMA) 2020 guidelines to ensure transparency and methodological rigor (Table S1).

One included study reported a mixed donor–patient cohort without donor-only breakdown for HBsAg/anti-HBc; we retained it with explicit labeling and evaluated its influence via leave-one-out, influence diagnostics, and design-adjusted models.

### 2.2. Eligibility Criteria

Studies were eligible if they: (1) reported original data on the prevalence of anti-HBc, HBsAg, and OBI among blood donors in Saudi Arabia; (2) were observational in design (cross-sectional, retrospective, or surveillance-based); (3) were published between January 2013 and April 2024; and (4) were studies published in English. Exclusion criteria included lack of extractable data, study populations other than blood donors, publication types such as reviews, case reports, editorials, or commentaries, or studies spanning different regions of the country.

### 2.3. Search Strategy

**Figure 1:**
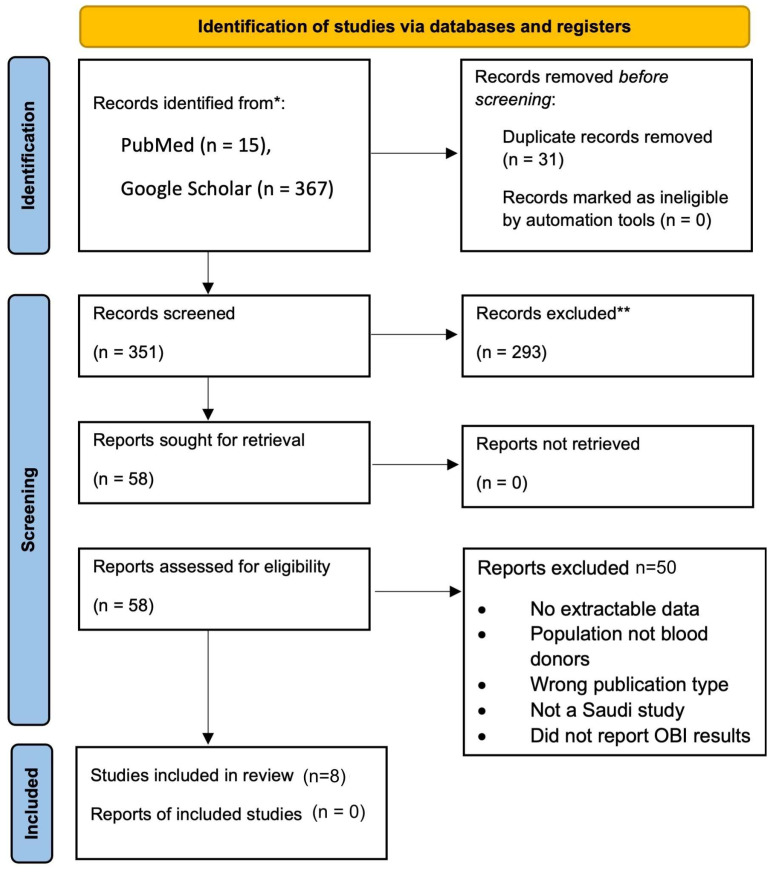
PRISMA 2020 Flow Diagram of Study Selection for the Meta-Analysis of Occult Hepatitis B Infection (OBI) Among Blood Donors in Saudi Arabia.

A comprehensive literature search was performed using MEDLINE (PubMed) and Google Scholar to identify relevant studies published between January 2013 and April 2024, as shown in Figure 1. A total of 15 records were retrieved from PubMed and 367 from Google Scholar. For Google Scholar, we screened only the first 200 results as a pre-specified feasibility constraint. After removing 31 duplicates, 184 unique records were retained. Additional studies were identified through manual citation tracking. After title and abstract screening of 184 unique records, 58 records were retained for full-text screening. Eight studies (Table 1) met the inclusion criteria and were included in the analysis.

### 2.4. Data Extraction

All records retrieved from the literature search were imported into Microsoft Excel, where duplicate entries were identified and removed. The author and the acknowledged researcher independently screened titles and abstracts, followed by full-text reviews of potentially relevant articles. Data from the included studies were extracted using a standardized Excel form. Extracted variables included first author, year of publication, study region, study design, data collection period, sample size, and the number of donors positive for HBsAg, anti-HBc, OBI, and HBV NAT. All extracted data were reviewed for completeness and consistency prior to meta-analysis.

### 2.5. Risk of Bias Assessment

To evaluate the methodological quality and potential risk of bias in the included studies, we applied the Joanna Briggs Institute (JBI) Critical Appraisal Checklist for Prevalence Studies [30]. This tool assesses key methodological domains relevant to observational studies estimating prevalence, including sampling strategies, sample size adequacy, measurement methods, and data analysis. Each of the nine included studies was independently appraised across the following nine criteria:

1. Appropriateness of the sample frame
2. Appropriateness of participant sampling
3. Adequacy of sample size
4. Clarity of subject and setting descriptions
5. Sufficient coverage of the sample
6. Validity of condition identification methods: OBI detection depends on the analytical sensitivity/specificity of both HBsAg and HBV-DNA NAT assays. Greater HBsAg sensitivity (lower IU/mL Limit of Detection (LOD)) can reduce the OBI pool (reclassifying some cases as HBsAg-positive), whereas greater NAT sensitivity increases OBI detection. For each study, we abstracted the HBsAg assay/platform and HBV-DNA NAT platform, and recorded HBsAg LOD, NAT format (ID vs. mini-pool), and HBV-DNA LOD (IU/mL) when reported (Table 1). Because most studies did not report one or more of these parameters, applying LOD-based inclusion/exclusion thresholds and performing LOD-restricted sensitivity analyses were not possible.
7. Consistency and reliability of measurement
8. Appropriateness of statistical analysis
9. Response rate or management of non-response

Each criterion was rated as “Yes,” “No,” “Unclear,” or “Not Applicable.” Studies were judged to have an overall low risk of bias if the majority of applicable domains were assessed as “Yes.” The assessment was based on a full-text review of each article.

### 2.6. Statistical Analysis

All statistical analyses were conducted in R (version 4.5.0) using the meta and metafor package v.4.8.0 [31]. Prevalence estimates for anti-HBc, HBsAg, and OBI were pooled using a random-effects model with logit transformation (measure = “PLO”) and the Hartung–Knapp adjustment to generate more accurate confidence intervals. Although generalized linear mixed models (GLMMs) are often recommended for rare-event data, a random-effects model was chosen to allow integration of prediction intervals, meta-regression, and publication bias diagnostics—features not fully supported in standard GLMM routines. Heterogeneity across studies was assessed using Cochran’s Q statistic, the I^2^ statistic, and the between-study variance (τ^2^). I^2^ values were interpreted using conventional thresholds: low (<25%), moderate (25–75%), and high (>75%). Leave-one-out sensitivity analyses were conducted using the metainf() function from the meta package v.8.2.0. These analyses evaluated the influence of each study on the overall pooled prevalence. Results were visualized using leave-one-out forest plots to determine whether any individual study had an undue influence on the overall findings.

### 2.7. Meta-Analysis Procedures

Separate meta-analyses were performed for each of the three HBV markers: anti-HBc, HBsAg, and OBI. Both fixed-effect and random-effects models were computed; however, interpretation focused on the random-effects results due to substantial heterogeneity across studies (I^2^ > 95%). Forest plots were used to visualize pooled and individual study-level prevalence estimates. Subgroup analyses by geographic region were conducted to examine regional heterogeneity, and meta-regression analyses were performed with region and publication year as covariates to explore variability in effect estimates and time trends. For the OBI dataset, where zero-event studies were common, a continuity correction of 0.5 was applied to all studies to allow for appropriate modeling under logit transformation.

### 2.8. Assessment of Publication Bias

Publication bias was assessed using Egger’s regression test using the rma() function in the metafor package v.4.8.0, and the Trim-and-Fill method (Duval and Tweedie) was applied to estimate the number of potentially missing studies and provide adjusted prevalence estimates. Rosenthal’s Fail-safe N was calculated using the fsn() function with a logit-transformed meta-analytic model. Begg’s rank correlation test was applied only to the anti-HBc and HBsAg datasets, where zero-event studies were not present. For the OBI dataset, Begg’s test was not performed due to the high prevalence of zero-event studies, which can invalidate test assumptions. Although Egger’s and Begg’s tests are generally recommended for datasets with 10 studies, they were applied in this study with 9 studies per marker and interpreted cautiously.

## 3. Results

### 3.1. Study Selection

A comprehensive literature search was performed using PubMed and Google Scholar to identify relevant studies published between January 2013 and April 2024. 15 records were retrieved from PubMed and 367 from Google Scholar. For feasibility, only the first 200 results from Google Scholar were screened. After removing 31 duplicates, 184 unique records were retained. Additional studies were identified through manual citation tracking. After title and abstract screening of 184 unique records, 58 records were retained for full-text screening. Eight studies (Table 1) met the inclusion criteria and were included in the meta-analysis. Data were extracted using a standardized Microsoft Excel spreadsheet. Extracted variables included: first author, year of publication, geographic region of the study, sample size, number and proportion of donors testing positive for anti-HBc, HBsAg, and OBI, whether NAT for HBV DNA was performed, and study design (e.g., cross-sectional or retrospective).

### 3.2. Characteristics of the Eligible Articles

A total of eight studies published between 2013 and 2024 were included in the final meta-analysis (Table 1). These studies collectively analyzed OBI in 87,820 blood donors across multiple regions in Saudi Arabia, including Aseer, Riyadh, Al-Majma’ah, Dammam, Al-Baha, and Jazan. All included studies utilized either cross-sectional or retrospective designs and performed NAT for HBV DNA, which is essential for identifying OBI. On average, anti-HBc positivity was about 4.8% (random-effects pooled estimate) ranging from 2% in Riyadh to 9% in Al-Majma’ah [20,28]. HBsAg positivity ranged 0.28–1.03% across the different studies [24,29] (Table 1).

OBI—defined as HBsAg-negative with detectable HBV DNA—was rare overall; for example, Aseer reported 73/6698 (1.09%) [21]. Other studies ranged 0.00–0.32%, including0.32% [25], 0.20% [20], 0.06% [22,24], 0.05% [29]. and 0.00% in Al-Majma’ah and Jazan [23,28].

### 3.3. Risk of Bias

The risk of bias across the nine included studies was low. All studies employed appropriate sample frames and valid diagnostic methods for detecting hepatitis B virus markers (anti-HBc, HBsAg and, where applicable, HBV DNA for OBI confirmation). Although most studies used convenience sampling without randomization, all had large sample sizes and applied uniform diagnostic procedures across participants. Data analysis methods were appropriate in all cases, with clearly reported prevalence estimates and summary statistics. The criterion related to response rate was “Not Applicable” in all studies, as they were retrospective analyses of blood donor records.

### 3.4. Pooled Prevalence

**Figure 2:**
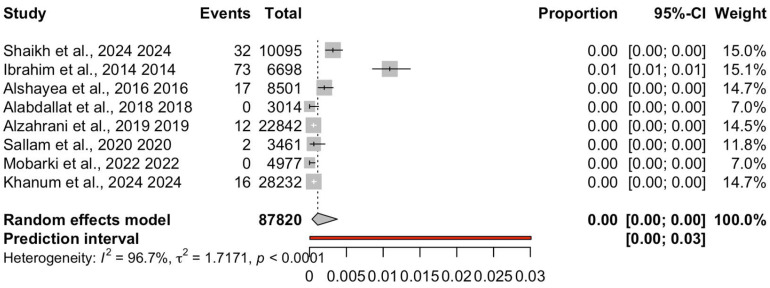
Forest plot of occult hepatitis B infection (OBI) prevalence among Saudi blood donors. Eight studies (k = 8 including [20,21,22,23,24,25,28,29]; N = 87,820; events = 152) were pooled using a random-effects model with logit transformation and Hartung–Knapp adjustment. The pooled prevalence was 0.12% (95% CI: 0.03–0.39%), with substantial heterogeneity (I^2^ = 96.7%, τ^2^ = 1.7171, *p* < 0.0001). Two studies reported zero events; a single higher-estimate study contributed disproportionately to heterogeneity.

The pooled prevalence estimates under the random-effects model were 5% for anti-HBc (95% CI: 3–7%), 0.46% for HBsAg (95% CI: 0.31–0.69%) and 0.12% for OBI (95% CI: 0.03–0.39%), Table 2. Fixed-effect results were directionally similar; interpretation focuses on the random-effects estimates given the substantial heterogeneity (I^2^ > 95%). These pooled estimates were computed using logit-transformed proportions with Hartung–Knapp adjustment to ensure conservative confidence intervals. Both anti-HBc and HBsAg prevalence were consistently reported across all included studies, permitting a direct comparison of past versus current HBV infection. The pooled prevalence of anti-HBc was 5% (logit = −2.94), while that of HBsAg was 0.46% (logit = −5.538), indicating that prior exposure (anti-HBc positive) was more common than active infection (HBsAg positive) among Saudi blood donors. For OBI, nine studies (N = 87,820; 152 OBI-positive) contributed to the synthesis; study-level estimates ranged from 0.00% (several zero-event studies) to 1.09%, as shown in Figure 2.

### 3.5. Heterogeneity

Marked between-study heterogeneity was observed for all three markers. For anti-HBc, heterogeneity was extremely high (I^2^ = 98.9%, τ^2^ = 0.2669, Q = 638.29, *p* < 0.0001), with a prediction interval ranging from 1.36% to 15.59%. Heterogeneity for HBsAg was also substantial (I^2^ = 90.6%, τ^2^ = 0.2068, Q = 74.23, *p* < 0.0001), and the prediction interval indicated possible variation between 0.15% and 1.44% in future comparable studies. Similarly, OBI demonstrated very high heterogeneity (I^2^ = 96.7%, τ^2^ = 1.7171, Q = 213.18, *p* < 0.0001), with a wide prediction interval of 0.00% to 3.0%; see Table 2. The particularly wide prediction intervals for HBsAg and OBI highlight variability across study populations and settings, including diagnostic and donor screening differences.

### 3.6. Meta-Analytic Parameters

Multiple assessments were conducted to evaluate the robustness, consistency, and potential biases of the pooled prevalence estimates; see Table 2. Trim-and-fill analysis imputed potentially missing studies in all three datasets. For anti-HBc, the trim-and-fill-adjusted prevalence was slightly lower than the primary random-effects estimate at 3.94% (95% CI: 2.54–6.08%), whereas for HBsAg and OBI the adjusted prevalence increased to 0.49% (95% CI: 0.33–0.73%) and 0.56% (95% CI: 0.11–2.87%), respectively. This pattern suggests that small-study effects and potential publication bias may have led to some underestimation of the true prevalence for HBsAg and, more notably, for OBI, while the impact on anti-HBc appears limited. Publication bias was evaluated using Egger’s regression and funnel plots. Egger’s test did not return valid *p*-values for anti-HBc, HBsAg, or OBI, reflecting the small number of studies (k = 8 for each marker) and the presence of zero-event and sparse-event data, so formal tests of funnel-plot asymmetry were inconclusive. In this context, greater weight was placed on graphical assessment and trim-and-fill results. As noted above, trim-and-fill suggested a modest downward adjustment for anti-HBc and upward adjustments for HBsAg and OBI, compatible with limited small-study effects rather than substantial bias.

Influence diagnostics indicated that the mixed-cohort study contributed to heterogeneity; however, pooled estimates were directionally similar in leave-one-out analyses. When each study was omitted in turn, the pooled anti-HBc prevalence remained between 4% and 5%, HBsAg between 0.4% and 0.6%, and OBI between 0.1% and 0.5%, with τ^2^ values changing only minimally across runs. This pattern indicates that the overall conclusions were not driven by any single study.

To further assess robustness to potential unpublished null studies, Rosenthal’s Fail-safe N was calculated on the logit-transformed prevalence estimates. Sensitivity analyses using a leave-one-out approach demonstrated that no individual study unduly influenced the overall pooled prevalence estimates. This reinforces the robustness and stability of the findings. Further, Rosenthal’s Fail-safe N calculations showed that 286, 950, and 164 null-result studies would be required to overturn the pooled effects for anti-HBc, HBsAg, and OBI, respectively, well beyond conventional thresholds, supporting the credibility and statistical resilience of the meta-analysis.

### 3.7. Subgroup Analysis by Region

For anti-HBc, the highest prevalence was observed in Al-Majma’ah (8.8%), while Riyadh (2.3%) was lowest (Table 1). Other regions such as Aseer, Al-Baha, the Eastern Province, and Jazan showed intermediate anti-HBc prevalences that broadly clustered around the overall pooled estimate, with overlapping confidence intervals in the regional subgroup forest plot.

For HBsAg, donor-only estimates were ≤~1.03% across regions, and most regions reporting values well below 1%. OBI remained low across regions, ranging from 0.00 to 1.09%, with the upper value observed in Aseer (73/6698). Despite these low absolute prevalences, confidence intervals were wide and substantial residual heterogeneity persisted in regional subgroup analyses.

### 3.8. Meta-Regression by Publication Year

Meta-regression analyses were conducted to assess the influence of publication year on prevalence estimates; see Table 3. The residual heterogeneity remained high in the models for anti-HBc (τ^2^ = 0.31, I^2^ = 99.2%, R^2^ = 0%), HBsAg (τ^2^ = 0.18, I^2^ = 88.0%, R^2^ = 12.0%), and for OBI (τ^2^ = 1.52, I^2^ = 94.4%, R^2^ = 8.0%). While the covariate region explained a substantial proportion of variability in the models for anti-HBc (R^2^ = 82.3%) and HBsAg (R^2^ = 98.9%), the effect of publication year was not statistically significant for any marker (*p* = 0.93 for anti-HBc; *p* = 0.25 for HBsAg; *p* = 0.28 for OBI).

### 3.9. Meta-Regression by Region

Separate meta-regression models were fitted with region as a categorical covariate (Table 3). For anti-HBc, the regional model yielded residual heterogeneity of τ^2^ = 0.11 and I^2^ = 98.3%, with region explaining R^2^ = 57.7% of the between-study variance; the omnibus test for regional differences was not statistically significant (QM = 2.86, *p* = 0.28; k = 8). For HBsAg, residual heterogeneity remained high (τ^2^ = 0.27, I^2^ = 95.0%), the proportion of variance explained by region was R^2^ = 0%, and the omnibus test was non-significant (QM = 0.67, *p* = 0.69; k = 8). For OBI, the regional meta-regression also showed high residual heterogeneity (τ^2^ = 2.14, I^2^ = 98.6%) with R^2^ = 0% and a non-significant omnibus test (QM = 0.78, *p* = 0.65; k = 8). All regional meta-regression estimates are summarized in Table 3.

## 4. Discussion

### 4.1. HBV Markers

This meta-analysis demonstrates a distinct serological profile among Saudi blood donors, where past HBV exposure is markedly more common than current infection. The anti-HBc to HBsAg prevalence ratio of approximately 8:1 indicates that the vast majority of seropositive donors had prior exposure to HBV that was subsequently resolved. This pattern is consistent with populations undergoing epidemiological transition as a result of the longstanding vaccination programs initiated in Saudi Arabia in early 1990s which led to decline in endemicity [24,32,33,34]. Where age information was available in the included studies, HBV markers and HBV-DNA positivity tended to increase with age, consistent with a pre-vaccination effect; however, OBI-specific ages were rarely reported, preventing pooled age analysis. Although the pooled HBsAg prevalence was low (0.46%), its presence indicates active infection persists in a small fraction of donors. In contrast, anti-HBc was detected in 5% of donors, signifying previous exposure.

Substantial between-study heterogeneity (I^2^ = 90.6–98.9%) was evident across all markers, reflecting genuine regional differences, variable screening practices, and potential demographic disparities; for example, Al-Majma’ah reported the highest anti-HBc and HBsAg prevalence, while Riyadh and Eastern Province showed the lowest. Nevertheless, sensitivity analyses supported the robustness of pooled estimates. Publication bias diagnostics suggested small-study effects for HBsAg and OBI, but these did not materially change the direction or magnitude of the pooled results.

Across genotype-D-dominant neighboring countries, observed OBI varied significantly. In Egypt, applying NAT to HBsAg-negative, anti-HBc-positive donors yields 10–18% OBI [35,36]; while in Lebanon, managing anti-HBc-reactive donors by deferral is associated with 0.3% among those tested [37]. In Iran, OBI estimates are 0.06% among all HBsAg negative donors but 8.81% in the anti-HBc-positive donors [38]. Our Saudi pooled prevalence OBI (0.012%) aligns with Iran’s overall estimate (0.06%), but is far below data reported from Egypt and Lebanon. The variations in OBI reporting support the interpretation that factors other than genotype are the main cause of these differences. Principal contributors include screening practice and assay sensitivity, donor-management policy, underlying endemicity, and the implementation and coverage of vaccination programs. To further explore temporal trends, meta-regression by publication year was performed. The results revealed no statistically significant effect of publication year on the prevalence estimates of any HBV marker (*p* =0.32 for anti-HBc; *p* = 0.149 for HBsAg; *p* = 0.392 for OBI), suggesting that seroprevalence rates have remained relatively stable over time, despite expectations of a decline post-vaccination. Notably, residual heterogeneity remained high in the anti-HBc and OBI models (I^2^ > 96%), indicating that temporal factors alone could not account for variability across studies. In contrast, regional differences explained a substantial proportion of variability, particularly for anti-HBc (R^2^ = 82.3%) and HBsAg (R^2^ = 98.9%). These findings reinforce the interpretation that geographic variability, rather than year of publication, remains the primary driver of prevalence differences, and emphasize the need for region-specific surveillance and screening strategies.

The lack of a significant temporal decline in HBV prevalence across the included studies may be attributed to several factors. First, the meta-analysis was limited to a specific subpopulation (blood donors) who are typically healthier and may not reflect broader community trends. Second, only studies that reported OBI results were included, which may introduce selection bias and possibly limiting the representativeness of earlier time periods. Third, the study period spanned only a single decade (2013–2024), which may not be sufficient to capture long-term epidemiological shifts.

### 4.2. OBI and Blood Safety

This meta-analysis confirms the presence of OBI among Saudi blood donors, with a pooled prevalence of 0.12% for OBI (95% CI: 0.03–0.39%), Globally, OBI prevalence among HBsAg-negative blood donors is estimated at approximately 0.2%, rising to 6.2% among those who are anti-HBc positive. These findings indicate that Saudi Arabia’s OBI prevalence lies below the global average. However, variations in OBI reporting, with studies reported 0% OBI, is most likely due to assay performance variability. Higher effective HBV DNA limits of detection—including those caused by sample dilution in mini-pool NAT LOD (>20 IU/mL) would be expected to miss the very low-level viremia characteristic of OBI. On the other hand, more sensitive HBsAg assays may reclassify borderline cases as an obvious infection, further lowering observed OBI cases. Nevertheless, Saudi Arabia has made substantial advancements in blood safety, particularly with the 2008 nationwide mandated NAT for HBV, HCV, and HIV. This policy shift significantly enhanced detection sensitivity and contributed to the overall reduction in transfusion-transmitted infections, which is consistent with international hemovigilance experience where countries such as Japan effectively eliminated OBI-related transmissions, with residual risk largely confined to window-period donations [12].

### 4.3. Hemovigilance in Saudi Arabia

Although OBI is a silent but serious threat to transfusion safety, its potential to transmit HBV remains underestimated without structured surveillance. Large-scale donor data indicate that a substantial proportion of donations are confirmed only after enhanced algorithms, reflecting intermittent, low-level viremia typical of OBI [13]. These observations reinforce the need for standardized confirmation pathways, cautious donor re-entry criteria, and a patient-focused hemovigilance system that links donor screening results to post-transfusion outcomes.

In the Saudi context, transfusion monitoring largely relies on passive reporting and voluntary notifications, echoing the global trend where even high-resource countries struggle with uniform implementation of hemovigilance frameworks [34,39]. This has led to significant underreporting and poor data integration, undermining the development of a national blood safety strategy [40].

Further complicating matters are fragmented data systems and inadequate integration between blood banks and hospital records, which impede the traceability of TTIs. Bloch [41] emphasizes that in many low- and middle-income countries (LMICs), including those with substantial healthcare budgets, post-transfusion surveillance remains underdeveloped or altogether absent. This weakens the ability to demonstrate causality and clinical sequelae, which are critical for guiding national blood safety strategies.

Studies showed that legal mandates, trained personnel, and institutional coordination are vital components of successful hemovigilance implementation [42]. In Saudi Arabia, the absence of these foundational elements limits the ability to capture adverse transfusion outcomes, impedes the identification of emerging risks such as OBI, and ultimately compromises the formulation of data-driven transfusion policies.

To align with international standards and WHO recommendations, Saudi Arabia needs to invest in a national recipient hemovigilance program that integrates hospital information systems with centralized blood safety surveillance. Enhancing diagnostic sensitivity, enforcing mandatory TTI reporting, and providing technician training on transfusion risk recognition. Bridging the gap between donor and recipient data is essential for advancing evidence-based transfusion policy in the Kingdom.

### 4.4. Strengths and Limitations

This study is the first to systematically examine OBI prevalence among blood donors in Saudi Arabia. By encompassing over 100,000 donations across diverse regions, it provides a foundational assessment of OBI in the national donor population. The integration of both serological (anti-HBc, HBsAg) and NAT markers enables a robust estimate of infection dynamics, offering valuable insights into residual transfusion risks. The use of advanced meta-analytic techniques, including random-effects models with Hartung–Knapp adjustments and meta-regression, enhances the accuracy and clarity of pooled prevalence estimates.

Despite these strengths, the study has several limitations. The relatively small number of included studies (*n* = 8) may constrain the generalizability of findings. We retained one mixed-cohort report as an annotated exception; its influence was assessed and did not materially alter the overall direction of pooled estimates.

Considerable heterogeneity in prevalence estimates was observed, likely stemming from differences in geographic region, donor populations, NAT methodologies, and study publication years. Egger’s test suggested small-study effects for HBsAg and OBI; these signals warrant caution—particularly given k < 10 and zero-event studies—but the direction and magnitude of pooled estimates were robust across sensitivity checks (leave-one-out, influence diagnostics, and trim-and-fill). These factors can distort regression-based assessments and reduce the power and validity of asymmetry tests in rare-event meta-analyses. Reporting gaps also limited our ability to conduct further analysis. Most studies did not report LOD or OBI-specific ages, and several did not specify whether NAT was ID or mini-pool. As a result, we could not predefine an LOD cutoff or conduct LOD-restricted analyses, and differences in pooled NAT versus identification-NAT (ID-NAT) likely influenced detection yield.

The certainty of evidence was not formally assessed using tools such as GRADE, due to the small number of included studies. However, consistency across studies and low risk of bias support confidence in the pooled estimates.

## 5. Conclusions

This meta-analysis provides the first comprehensive estimate of anti-HBc, HBsAg, and OBI prevalence among blood donors in Saudi Arabia. While the prevalence of active infection remains low, a significant proportion of donors have serological evidence of past HBV exposure, and a small but clinically important subset harbor OBI detectable only by NAT. These findings underscore the importance of maintaining high standards in donor screening, including the integration of NAT and anti-HBc testing to mitigate residual risk. Public health strategies should be continually evaluated to adapt to changing HBV epidemiology and ensure transfusion safety. Broader implementation of targeted regional interventions and national donor registries may support efforts to eliminate transfusion-transmitted HBV and strengthen Saudi Arabia’s blood transfusion safety infrastructure.

## Figures and Tables

**Table 1:** Summary of Included Studies Reporting Serological and Molecular Markers of HBV Among Blood Donors in Saudi Arabia.

Study	Year	Region	Data Collection Period	Study Design	Sample Size	Anti-HBc (%)	HBsAg (%)	OBI (%)	NAT Method	HBsAg LOD (IU/mL)	HBV DNA LOD (IU/mL)
Shaikh et al., 2024 [25]	2024	Aseer	2020–2022	Retrospective, single-center	10,095	443 (4.39)	37 (0.37)	32 (0.32)	NR	NR	NR
Ibrahim et al., 2014 [21]	2014	Aseer	2012–2013	Cross-sectional	6698	411 (6.14)	69 (1.03)	73 (1.09)	NR	NR	NR
Alshayea et al., 2016 [20]	2016	Riyadh	2016	Cross-sectional	8501	198 (2.33)	56 (0.66)	17 (0.20)	ID-NAT	NR	16.4
Alabdallat et al., 2018 [28]	2018	Al-Majma’ah	2018	Cross-sectional, single-center	3014	264 (8.76)	8 (0.27)	0.00	ID-NAT	NR	NR
Alzahrani et al., 2019 [29]	2019	Dammam	2019	Retrospective	22,842	664 (2.91)	63 (0.28)	12 (0.05)	ID-NAT	NR	NR
Sallam et al., 2020 [24]	2020	Al-Baha	2020	Retrospective cross-sectional	3461	253 (7.31)	10 (0.29)	2 (0.06)	NR	NR	NR
Mobarki et al., 2022 [23]	2022	Jazan	2022	Retrospective	4977	364 (7.31)	30 (0.60)	0.00	ID-NAT	NR	NR
Khanum et al., 2024 [22]	2024	Aseer	2024	Retrospective, multi-center	28,232	908 (3.22)	133 (0.47)	16 (0.06)	NR	NR	NR

LOD = Limit of Detection; NR = not reported; ID-NAT = Identification NAT.

**Table 2:** Comparative summary of pooled prevalence, heterogeneity, and publication bias diagnostics for anti-HBc, HBsAg, and OBI among Saudi blood donors (logit-transformed proportions; Hartung–Knapp).

Statistic	Anti-HBc	HBsAg	OBI
Fixed-Effect Estimate [95% CI]	0.0538 [0.0524–0.0553]	0.00486 [0.00465–0.00507]	0.0038 [0.0032–0.0044]
Random-Effects Estimates (PLO + HK) [95% CI]	0.0517 [0.0348–0.0760]	0.0046 [0.0031–0.0069]	0.0012 [0.0003–0.0039]
95% prediction interval	[0.0136–0.1559]	[0.0015–0.0144]	[0.0000–0.0299]
Heterogeneity: I^2^	98.9%	90.6%	96.7%
Heterogeneity: τ^2^	0.2669	0.2068	1.7171
Heterogeneity: Q (df, *p*)	638.29 (8, <0.0001)	74.23 (7, <0.0001)	213.85 (7, <0.0001)
Trim-and-Fill adjusted prevalence [95% CI]	0.0394 (0.0254–0.0608)	0.0049 (0.0033–0.0073)	0.0057 (0.0011–0.0282)
Fail-safe N (Rosenthal)	386	950	164

**Table 3:** Meta-Regression Results by Year and Region.

	Meta-Regression by Publication Year			
Marker	Residual I^2^ (%)	τ^2^ (Residual)	R^2^%	*p*-Value
Anti-HBc	99.2	0.31	0%	0.93
HBsAg	88	0.18	12%	0.25
OBI	94.4	1.52	0%	0.28
	Meta-regression by publication region			
Anti-HBc	98.3	0.11	57.7	0.28
HBsAg	95	0.27	0	0.69
OBI	98.6	2.14	0	0.65

anti-HBc, antibody to hepatitis B core antigen; HBsAg, hepatitis B surface antigen; OBI, occult hepatitis B infection; τ^2^, between-study variance (residual heterogeneity); I^2^, percentage of total variation due to heterogeneity after including the moderator; R^2^, proportion of between-study variance explained by the moderator.

## Data Availability

All data analyzed in this study were obtained from published sources, as detailed in the manuscript. The datasets supporting the conclusions are available from the corresponding publications cited in the reference list. No new raw data were generated or analyzed specifically for this study.

## Supplementary Materials

The following supporting information can be downloaded at: https://www.mdpi.com/article/10.3390/diagnostics15233004/s1, Table S1: PRISMA 2020 Checklist. Reference [43] is cited in the supplementary materials.
